# Specialized Laparoscopic Devices in the Treatment of Hydatic Hepatic Cysts: A Retrospective Analysis and Review of the Literature

**DOI:** 10.7759/cureus.55968

**Published:** 2024-03-11

**Authors:** Alin Mihetiu, Dan Georgian Bratu, Alexandra Sandu, Alexandru Sabau, Dan Sabau

**Affiliations:** 1 Second Surgical Department, "Lucian Blaga" University of Sibiu, County Clinical Emergency Hospital of Sibiu, Sibiu, ROU; 2 Faculty of Medicine, "Lucian Blaga" University of Sibiu, Sibiu, ROU

**Keywords:** hepatic echinococcosis, hydatid cyst, scolicidal agents, laparoscopic approach, hydatidosis

## Abstract

Background and objective

While hydatid disease is associated with a high prevalence only in certain endemic areas, it can be encountered in any geographical region. The characteristics of this parasitic disease, and its implications during development, such as the risk of seeding, and the complications caused by cyst rupture, means that its therapeutic management should adhere to strict principles and may sometimes require approaches specially tailed for this type of pathology. In this study, we aimed to provide a comparative analysis of conventional laparoscopic techniques vs. treatment with specialized instrumentation in these patients.

Methods

Our study involved a retrospective evaluation of a cohort comprising 41 patients diagnosed with hepatic hydatid cysts, who underwent procedures with both conventional laparoscopic techniques and specialized instrumentation tailored for this particular pathology. Furthermore, we conducted a comprehensive review of the literature examining alternative types of laparoscopic instrumentation specifically crafted for the management of hydatid cysts. This review employed an extensive search utilizing PubMed and Google Scholar databases.

Results

The examination of cases within our study revealed a high prevalence of hydatid disease among male patients (63.41%) and a predominance of instances originating from rural regions necessitating emergent admissions (p<0.05). Notably, in 58.54% of cases, surgical interventions employed specialized instrumentation, with a notable discrepancy in conversion rates to open surgery favoring the standard approach: 12.2% vs. 2.44% (p=0.025). Additionally, the laparoscopic approach was associated with prolonged surgical durations compared to the dedicated technique (p=0.002), besides a higher incidence of postoperative complications (12.2% vs 7.32%). Furthermore, patients undergoing laparoscopic procedures with standard instrumentation experienced lengthier hospital stays (p=0.002).

Our comprehensive review of the literature identified six distinct surgical methodologies utilizing specifically tailored instrumentation for addressing hydatid cysts. Analysis of these findings underscored a preference for single localizations and selective cases. Postoperative complication rates ranged from 6.66% to 22.22%, with conversion rates to open surgery reaching up to 23.33%, and recurrence rates observed to be as high as 7.81%.

Conclusions

The patented approach, which uses special trocars that provide stable anchorage and allow a safe puncture-aspiration, reaspiration, and fragmentation processes, has superior characteristics compared to the laparoscopic approach with standard instrumentation. Comparative analysis with other similar procedures described in the literature has shown similar results regarding the frequency of complications, with our technique being superior in terms of approaching multiple cysts and recurrence rate. It has been successfully applied even in unselected cases.

## Introduction

Hydatid disease has been known since ancient times when descriptions by Celsus, Aretaeus, and Galen were compiled in the Hippocratic Corpus [1]. The first therapeutic attempts were made in the mid-19th century by French surgeons such as Recamier and Moissenet, who practiced cyst puncture. At the end of the 19th century, Lagenbuch performed cyst puncture and aspiration followed by instillation with various substances such as iodine tincture, ox bile alcohol, and boric acid. The evolution was burdened by local and systemic complications and especially by recurrences.

In 1871, the first excision of the internal capsule of the hydatid cyst was performed, but cases were frequently associated with local recurrence, intra-abdominal implantations, or infections of the remaining cavity [2,3]. For a long time, the surgical approach to hepatic hydatid cysts did not undergo significant changes, with innovations mainly focusing on the treatment of cyst contents and the management of the cyst wall and residual cavity. Surgical interventions primarily involved open surgery. In 1992, Seven et al. reported the first laparoscopic resolution of the hydatid cyst; since then, this approach has become the main method of surgical management of this pathology, with robotic treatment being the latest addition [4].

The first percutaneous drainage under radiological guidance, based on the principles of puncture, aspiration, instillation, and reaspiration (PAIR), was performed in 1988; the first study on a significant group of patients was reported in 1991 [5]. PAIR has subsequently become the predominant method in the management of hydatid cysts, surpassing the open surgery approach, and has come to be preferred over laparoscopy as a superior minimally invasive method in certain aspects. Until the introduction of PAIR as the first-line invasive therapeutic approach, laparoscopy constituted the only minimally invasive method for this pathology. In contrast to open surgery, the laparoscopic approach has significant advantages in terms of postoperative complications, recovery, recurrence, and costs related to medical assistance and socio-professional reintegration.

A pivotal concern in the invasive management of hydatid cysts revolves around the potential for intraoperative rupture and dissemination of cyst contents. The contents of the cyst harbor significant allergenic properties, which, depending on the patient's immunological profile, may precipitate anaphylactic shock. Allergic or anaphylactic reactions manifest as immune responses mediated by IgE hypersensitivity, wherein specific Echinococcus antigens bind to mast cells or basophils via Fc receptors. Subsequently, this binding triggers the release of mediators such as histamine, enzymes, and lipid mediators into the bloodstream [6]. Most commonly, these allergic/anaphylactic complications result from spillage during invasive procedures, although they can also occur more rarely and spontaneously. The risk of intraoperative anaphylaxis is estimated at 1/20.000 procedures, with a mortality rate ranging from 3 to 6%. Apart from triggering the cascade of anaphylactic shock, anesthetic medication can also contribute to accentuating these phenomena [7].

Awareness of the increased risk associated with this certain medication (e.g., neuromuscular blocking agents such as suxamethonium, atracurium, and rocurium) is essential. Hence, it is necessary to establish surveillance programs to prevent, identify, monitor, and manage adverse drug reactions (ADRs). To ensure that best practices are followed, personalized dosing strategies should be employed, patient characteristics should be considered, and a multidisciplinary approach should be adopted [8]. Preferably, the management of these allergic or anaphylactic reactions during a radiological-surgical procedure aims to mitigate the occurrence of spontaneous anaphylaxis. However, despite meticulous adherence to safety protocols, the risk of intraoperative spillage or during PAIR procedures remains considerable, with documented rates ranging from 5 to 10% [9]. Intraperitoneal seeding due to cyst breach leads not only to allergic reactions or anaphylaxis but also to cyst recurrence with peritoneal hydatidosis. Such a situation significantly worsens the patient's prognosis, exposing them to the risk of surgical reintervention. The evolutionary stage of the hydatid cyst and its variety of locations have necessitated the standardization of a therapeutic algorithm [10].

The predominant pharmacological agent employed in the treatment of hydatid cysts is albendazole, although its efficacy is reported to be inferior to that of praziquantel or a combination of both medications. Furthermore, while drug therapy offers certain benefits in the management of hydatid cysts, its effectiveness is constrained by factors such as the size and location of the cysts, as well as the necessity for prolonged treatment regimens. Nonetheless, the value of pharmacological treatment is undeniable, particularly as a preparatory measure preceding or associated with invasive interventions [11]. The scolicidal substances used in PAIR and surgery are the same, with each substance varying in terms of the degree of parasiticidal action and its behavior towards hepatic parenchyma, the biliary tree, or neighboring tissues.

Due to frequent complications such as hepatic parenchymal necrosis, cholangitis, biliary cirrhosis, and acute liver failure, most scolicidal agents have been removed from current practice. The WHO recommends the use of the hypertonic saline solution, alcohol, or povidone-iodine, but even these have limitations regarding inactivation or possible complications [10]. To avoid the risk of systemic immune reactions and abdominal contamination, it is necessary for the invasive approach to achieve inactivation of the cyst contents, and for the connection between the trocar and the cyst wall to be airtight during the puncture, aspiration, and reaspiration stages. The standard laparoscopic approach, although it can achieve isolation with a gauze soaked in a scolicidal agent, cannot prevent the externalization of hydatid fluid during the primary puncture or other maneuvers. Thus, various instruments or adaptations of standard laparoscopic instruments have been envisaged and designed, which will be analyzed alongside our method in the following sections.

## Materials and methods

Study design

We conducted a dual analysis, comprising a retrospective study centered on our clinical encounters pertaining to the management of hepatic hydatid cysts utilizing specialized instrumentation, alongside a comprehensive literature review encompassing techniques tailored to the distinctive attributes of hydatid cysts. In the review, articles analyzing the approach toward hepatic hydatid cysts using specially designed instruments for this type of pathology were searched on PubMed and Google Scholar databases. In the retrospective study, patients from the Second Surgical Department of Emergency County Hospital of Sibiu with hepatic hydatid cysts who underwent a laparoscopic approach using both standard instrumentation and dedicated instrumentation were analyzed. The study period spanned five years (January 2018 - January 2022), during which 41 patients were identified.

The study included patients with exclusively hepatic hydatid cysts, regardless of the number of cysts, and only those patients who underwent a laparoscopic were studied. Patients for whom open surgery was preferred as the first-line approach were not included in the study. Surgical techniques consisted of the standard laparoscopic approach to the upper abdominal floor (using standard laparoscopic instrumentation and adapting the positioning and number of trocars according to the location of the cysts) and laparoscopic instrumentation specially designed and adapted for such pathology.

Surgical technique

The study involving the technical approach adopted in our surgical department is based on specially designed instrumentation aimed at fulfilling the requirements of a minimally invasive and safe approach to the hydatid cyst. The instrumentation for hydatid cysts by Dan Sabau (State Office for Inventions and Trademarks Patent No. 120809/30.04.2008) represents an adaptation of standard laparoscopic instrumentation to the intraoperative needs related to the characteristics of the cyst wall and contents.

The laparoscopic approach begins with standard pneumoperitoneum creation up to 12 mmHg, with a 10 mm umbilical trocar being inserted. The placement of the other trocars is adjusted according to the localization of the hydatid cyst. The 20 mm trocar is inserted and fixed to the cyst wall, with the aspiration and instillation trocar subsequently achieving stable anchoring to the cyst wall using hooks. Since the two systems function coaxially and each has its own suction system, any leaks from the central trocar will be captured and aspirated by the second trocar. Additionally, it allows the instillation of a scolicidal agent (hypertonic saline or alcohol) through the central trocar and aspiration through the peripheral one. The anchoring is thus stable even during aspiration, instillation, or morselation maneuvers of the contents, and scopic exploration of the cyst cavity, creating a true working chamber, supplementary to the peritoneal one [12].

**Figure 1:**
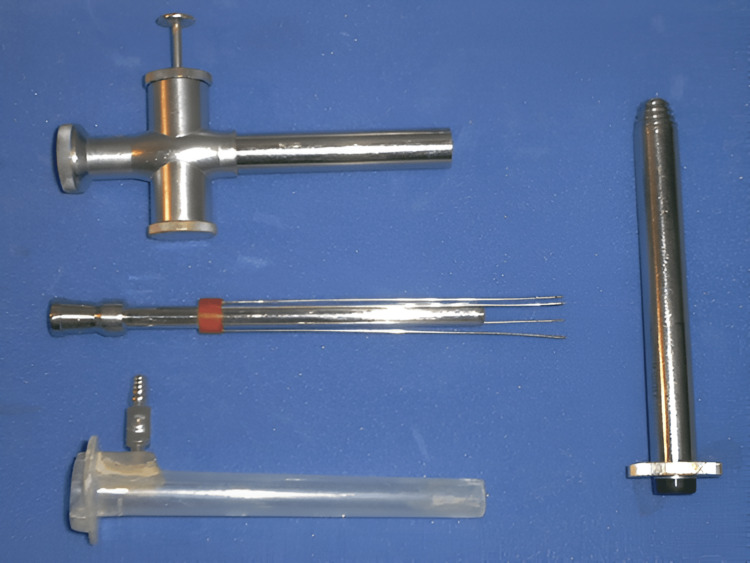
Devices for aspiration, reaspiration, and fragmenting cystic content

**Figure 2:**
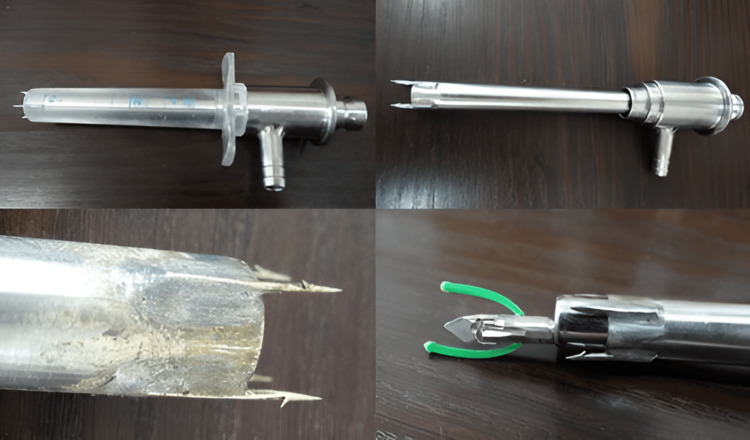
Instrumentation for aspiration, reaspiration, and fragmenting cystic content

The fragmentation device is intended to homogenize the contents of the cyst for more efficient aspiration. The instrumentation includes certain safety features in addition to the alcoholized field, allowing for the use and exchange of aspiration, instillation, reaspiration, or fragmentation instruments through an isolated working channel, thus preventing the risk of spillage and contamination through instrumentation or additional maneuvers (Figures 1, 2). This adaptation of the usual laparoscopic instrumentation has become the main approach to hydatid cysts in our service, surpassing the standard laparoscopic approach. The successful use of the device has been demonstrated in cases with multiple localizations, difficult localizations (segments IVB, V, cysts in contact with the gallbladder, portal vein, or major bile ducts), cases with concurrent liver-diaphragm-pleural involvement, or in pediatric cases [13,14].

Statistical analysis

The data were collected using MS Excel (Microsoft Corp., Redmond, WA) and analyzed using SPSS Statistics version 21.0 (IBM Corp., Armonk, NY), with additional statistical analysis performed using DATATab (DATAtab e.U., Graz, Austria).

Methodology for the literature review

The methodology employed for the review process involved a systematic approach, characterized by thorough database exploration and the meticulous application of predetermined exclusion criteria. We conducted a systematic review following the 2020 guidelines outlined in the Preferred Reporting Items for Systematic Reviews and Meta-Analyses (PRISMA) [11]. An exhaustive literature search was performed on PubMed and Google Scholar databases. The keywords used were “hydatid cyst’, “laparoscopy”, “special devices”, “modified laparoscopic instruments” and “ dedicated instruments”. The initial search of the two databases yielded 185 results: 67 from PubMed and 118 from Google Scholar. After applying the exclusion criteria, 11 publications were considered eligible.

Inclusion Criteria

For our research, we conducted a review of relevant studies without any restrictions related to the time of publication, focusing on articles in the English language. The inclusion criteria involved studies that describe the laparoscopic approach with instrumentation other than the standard one, and dedicated instrumentation for the surgical approach of the hydatid cyst used in single or multiple localizations of hepatic hydatid disease in adult patients or pediatric patients.

Exclusion Criteria

Case reports, case series, dissertations, book chapters, conference abstracts, letters to the editor, editorials, state-of-the-art articles, and technical reports without statistical analysis were excluded. Additionally, studies with insufficient data, duplicates, or articles that did not clearly present the type of technique and instrumentation used were also excluded. No systematic reviews or meta-analyses on the subject were identified.

Bias

**Figure 3:**
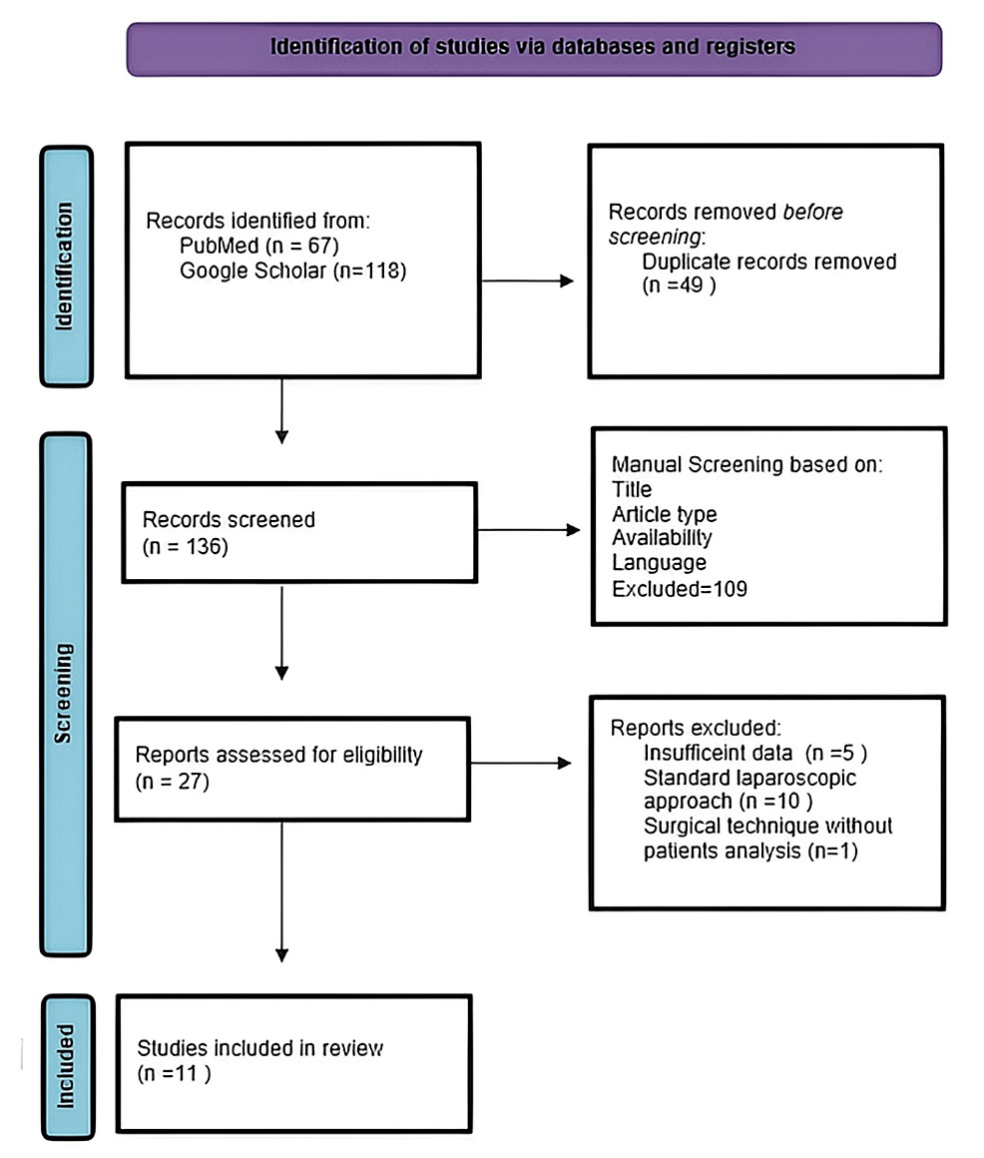
PRISMA chart illustrating the selection of articles PRISMA: Preferred Reporting Items for Systematic Reviews and Meta-Analyses

All articles included in this publication were assessed for bias. The only article type that could be considered as posing a risk of bias was that involving pediatric patients. Given the very small number of results that met the research criteria and the fact that the type of instrumentation used applies to both pediatric and adult patients, with the only difference being the pressure values of the working chamber (5-7 mmHg), we opted to include this type of articles in the analysis. The findings derived from our systematic review, following diligent database exploration and stringent application of exclusion criteria, have been consolidated in the Discussion section of our article. Figure 3 shows the PRISMA chart depicting the selection of the articles.

## Results

Patient characteristics

The mean age of the cohort was 49.34 ± 18.65 years (range: 4-81 years); 9.75% (n=4) of patients were in the pediatric age group (<18 years old). We identified a higher frequency of hydatid disease in males (63.41%) compared to females (36.59%), with a mean age of 52.69 ± 18.58 years for males and 43.53 ± 17.88 years for females. Patients with this condition were predominantly from rural areas (58.54%). A notable proportion of patients (24.39%) presented with emergent conditions, whereas 75.61% sought medical attention on an outpatient basis. The incidence of emergency hospital admissions was notably higher among patients from rural locales (21.95% vs. 2.44% from urban areas, p=0.013).

Preoperative parameters

The most commonly encountered symptom was abdominal pain, predominantly localized in the right hypochondrium (82.93%), followed by asthenia (14.63%) and nausea (2.44%). A total of 22 patients (53.66%) presented with a single hydatid, while 46.34% presented with multiple localizations of the disease. Of note, 60.98% of cases received preoperative therapy with albendazole 10 mg/kg/day, with 90% of those presenting as emergency cases not receiving antihelmintic therapy (p=0.003). The topographic distribution of hydatid cysts is shown in Table 1.

**Table 1:** Topographic distribution of hydatid cysts

Cyst number							
Solitary				Multiple			Total
Location (liver segments)	%	% within placement	% within cyst number	%	% within placement	% within cyst number	%
VII	23.08%	42.86%	100%	0%	0%	0%	23.08%
IV-V	2.56%	4.76%	100%	0%	0%	0%	2.56%
VI	5.13%	9.52%	100%	0%	0%	0%	5.13%
V, VI.VII	0%	0%	0%	2.56%	5.56%	100%	2.56%
VI, II	0%	0%	0%	2.56%	5.56%	100%	2.56%
V, VI, VII	0%	0%	0%	2.56%	5.56%	100%	2.56%
VIII, IV	0%	0%	0%	2.56%	5.56%	100%	2.56%
IV, VI	0%	0%	0%	2.56%	5.56%	100%	2.56%
V	2.56%	4.76%	100%	0%	0%	0%	2.56%
II, III	2.56%	4.76%	50%	2.56%	5.56%	50%	5.13%
VII, VIII	0%	0%	0%	2.56%	5.56%	100%	2.56%
VI, VII	2.56%	4.76%	16.67%	12.82%	27.78%	83.33%	15.38%
IV	5.13%	9.52%	66.67%	2.56%	5.56%	33.33%	7.69%
IV, V	2.56%	4.76%	100%	0%	0%	0%	2.56%
VIII	2.56%	4.76%	100%	0%	0%	0%	2.56%
IV, VII	0%	0%	0%	7.69%	16.67%	100%	7.69%
VI, VII, VIII	0%	0%	0%	2.56%	5.56%	100%	2.56%
II, IV, V	2.56%	4.76%	100%	0%	0%	0%	2.56%
III	2.56%	4.76%	100%	0%	0%	0%	2.56%
V, VII	0%	0%	0%	2.56%	5.56%	100%	2.56%
Total	53.85%	100%	-	46.15%	100%	-	100%

Surgical parameters

The analysis of surgical parameters revealed that the primary technical choice involved the utilization of dedicated instrumentation, with this method being adopted in 58.54% (n=24) of the cases included in the study. The standard laparoscopic approach was employed in 41.46% of patients. Intraoperative complications were not documented in 87.8% of cases, with the most common complication being the spillage of cyst contents, an occurrence exclusively observed in the standard laparoscopic approach. However, this complication did not achieve statistical significance (t=-1.66, p=0.105, d=0.53) when compared to the alternative approach.

Conversion to open surgery from the laparoscopic approach was required in 14.63% of cases, with such occurrences being less frequent in cases utilizing dedicated instrumentation for hydatid cysts in contrast to those undergoing standard laparoscopic management: 2.44% vs. 12.2% (t=-2.08, df=20.31, p=0.025, d=0.66). Conversions to open surgery were more prevalent in cases with multiple localizations (10%) compared to single localizations (5%). Notably, 50% (n=3) of conversions were attributed to challenging localizations, with two instances necessitating conversion due to diaphragmatic penetration resulting in abscess formation and free perforation into the peritoneum, and one case due to spillage during laparoscopic maneuvers in a technically challenging position.

**Figure 4:**
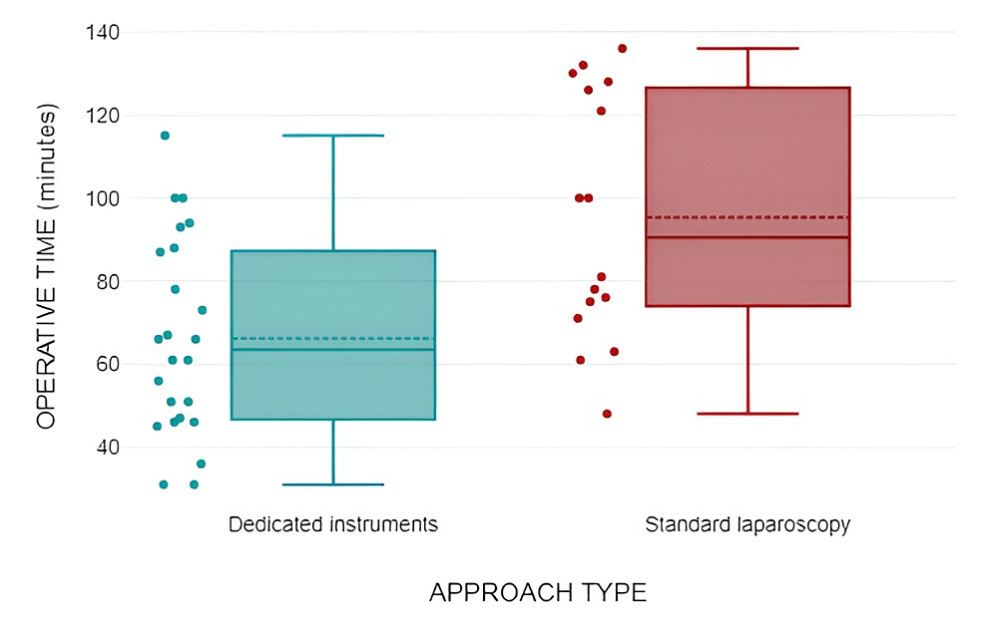
Correlation between surgical approach type and intraoperative time

The surgical approach to managing the hydatid cyst encompassed partial pericystectomy (58.54%), operculectomy (19.51%), pericystectomy (17.07%), and Lagrot procedure (for conversion) (4.88%). Partial pericystectomy represented the predominant option in both the approaches with dedicated instrumentation (57.17%) and the standard laparoscopic approach (64.71%). Additionally, the radical approach-total cystectomy was more frequently undertaken in cases employing dedicated instrumentation compared to the standard laparoscopic approach (25% vs. 5.88%), albeit without reaching statistical significance (p>0.05). The duration of surgical intervention was higher in the laparoscopic approach (93.41 ± 29.9 minutes) compared to the technique utilizing special instruments (66.21 ± 23.68 minutes), and the difference was statistically significant (Table 2, Figure 4).

**Table 2:** Statistical correlation between surgical approach and intraoperative time

Statistical correlation test	Correlation coefficients
t-test for independent samples	t=-3.25
	p=0.002
	d=1.03
	95% CID=-44.14
Mann-Whitney U test	z=-2.82
	Asymptotic p=0.005
	Exact p=0.005
	r=0.44
Spearman correlation analysis	r=0.45
	p=0.003

**Figure 5:**
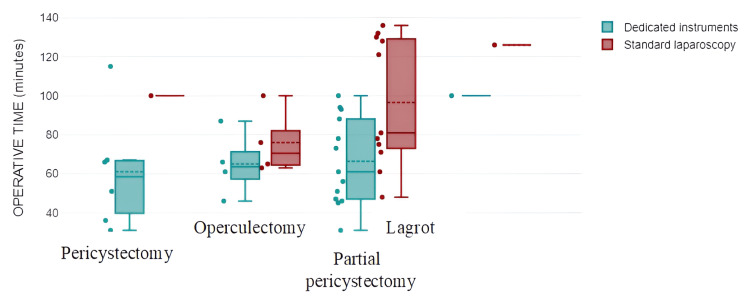
Chart representation of cyst management in relation to operative time

Upon scrutinizing the type of approach toward the cyst and correlating it with the duration of surgical intervention, it was discerned that the utilization of specially designated instrumentation resulted in a shorter operative duration, irrespective of the management modality of the cysts (Chi^2^=10.26, df=3, p=0.012) (Table 3, Figure 5).

**Table 3:** Operative time for different methods of cyst management

Surgical parameter	Type of approach	Surgical attitude towards the cyst	Frequency	Mean	Std. deviation	Minimum	Maximum
Operative time (minutes)	Dedicated instruments	Pericystectomy	6	61	30.34	31	115
		Partial pericystectomy	13	66.38	22.59	31	100
		Operculectomy	4	65	16.95	46	87
		Lagrot	1	100	NaN	100	100
	Standard laparoscopy	Pericystectomy	1	100	NaN	100	100
		Partial pericystectomy	11	96.45	32.93	48	136
		Operculectomy	4	76	16.99	63	100
		Lagrot	1	126	NaN	126	126

Postoperative parameters

The most frequent postoperative complication was bile leak (present in 19.51% of cases), followed by subcutaneous emphysema and hematoma (each 2.44%). Biliary fistula was more common in the group with the conventional approach (12.2% vs. 7.32%). However, the analysis of these parameters did not show statistical significance.

The most common complication (bile leak) was detected with a higher frequency in patients who underwent partial pericystectomy (9.76%) compared to the group with pericystectomy (7.32%). These values change when reporting the number of interventions (n=24 for partial pericystectomy and n=7 for pericystectomy). Thus, the frequency of biliary complications was higher in radical approaches (42.85%) compared to those preserving partial cyst wall at the intraparenchymal level (16.66%) and compared to operculectomy, without statistical significance regarding biliary complications when analyzing each type of cyst approach.

Patients admitted as emergency cases required a longer hospitalization compared to those admitted without acute symptoms (12.33 ± 4.12 days vs. 7.87 ± 3.4 days). Both urgent presentation and postoperative complications altered the duration of hospitalization, with a statistically significant correlation between these variables (Tables 4-5).

**Table 4:** Statistical analysis between emergency presentation and hospitalization

Statistical correlation test		Correlation coefficients
Pearson correlation	r=0.47	
	p=0.002	
t-test for paired samples	t=-10.66	
	df=39	
	p<0.001	
	d=1.69	
t-test for independent samples	t=11.18	
	df=40.92	
	p=<0.001	
	d=1.69	

**Table 5:** Statistical analysis between postoperative complications and hospitalization

Statistical correlation test	Correlation coefficients
Pearson correlation	r=0.41
	p=0.007
t-test for paired samples	t=-14.56
	df= 40
	p<0.001
	d=2.27
t-test for independent samples	t=-12.76
	df=47.49
	p<0.001
	d=2.82

**Figure 6:**
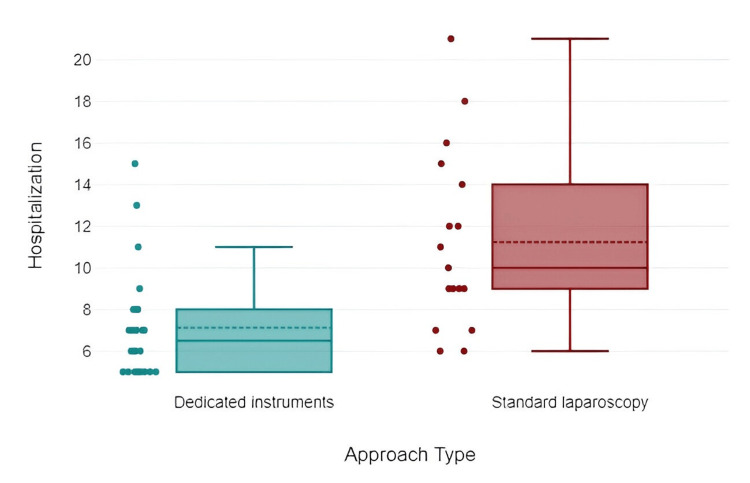
Chart representation of the impact of surgical approach on hospitalization

When examining the duration of hospital stays based on the surgical approach used, we noted that patients undergoing the standard laparoscopic procedure tended to have longer hospitalization periods (Table 6, Figure 6).

**Table 6:** Hospital stay in relation to surgical approach

Postoperative parameter	Type of approach	Mean	Std. deviation	Minimum	Maximum
Hospitalization length (days)	Dedicated instruments	7.13	2.63	5	15
	Standard laparoscopy	11.24	4.32	6	21

Analyzing these data, statistical significance was evident in the relationship between surgical technique and the duration of hospitalization (t=-3.49, p=0.002, d=1.11). Hospitalization was longer in patients undergoing partial pericystectomy (9.42 ± 4.49 days) compared to pericystectomy (7.86 ± 3.67 days) or operculectomy (7.5 ± 2.88 days). Statistical analysis demonstrated the following data: Pearson correlation: r=0.18, p=0.273; Spearman correlation: r=0.23, p=0.146; Chi^2^=2.3, p=0.512.

No cases of recurrence were detected, and late complications involved wound seroma and intraparenchymal collection, which were conservatively treated, and there was no statistical significance in terms of the type of approach or surgical management of the cyst (p>0.05).

## Discussion

The findings from our literature review have been synthesized in Table 7.

**Table 7:** Summary of the reviewed studies PGAA: Perforator Grinder-Aspirator Apparatus

Authors	Type of device	Number of patients	Number of cysts	Conversion	Cyst management	Intraoperative complication	Postoperative complications	Recurrence
Sormaz and Avtan [15]	PGAA	42	32 single, 10 multiple	2	Drainage, drainage, and unroofing	Bile duct injury (3)	Biliary fistula (1)	-
Kaya et al. [16]	Veress needle and 10 mm trocar	18	11 solitary, 7 multiple	1	Drainage and omentoplasty	-	Biliary fistula 2, pleural effusion, trocar site infection	-
Alper et al. [17]	PGAA	22	13 solitary, 3 multiple	6	Drainage 4, unroofing 12	-	Biliary fistula (2), cavity infection (1), abscess (1)	-
Hemmati [18]	Hemmati system	10	8 solitary,	0	Drainage, unroofing	-	-	-
Aslam et al. [19]	Palanivelu system	45	41 solitary, 4 multiple	0	Drainage, unroofing 40; drainage, left lobectomy 5	-	Biliary fistula 5, cavity infection 1	1
Palanivelu et al. [20]	Palanivelu system	66	60 solitary, 6 multiple	0	Drainage, unroofing; drainage, lobectomy 9; transcystic fenestration 2	-	Biliary fistula 9, cavity infection 2	-
Kanojia [21]	Port-in-cyst technique	6	5 solitary, 1 multiple	0	Drainage 6	-	-	-
Kayaalp [22]	Port-in-cyst technique	19	14 solitary, 5 multiple	0	Drainage 19	-	-	-
Palanivelu et al. [23]	Palanivelu system	75	68 solitary, 7 multiple	0	Evacuation and marsupialization 62, evacuation and lobectomy 10, transcystic fenestration 2	-	Biliary fistula 9, cavity infection 2	-
Senthilnathan et al. [24]	Palanivelu system	105	89 solitary, 16 multiple	0	Evacuation and cystectomy 91, evacuation and left lateral segmentectomy 10, total pericystectomy 1, others 3	-	Biliary fistula 13, cavity infection 2, duodenal injury 1	2
Seven et al. [4]	Umbrella trocar	30	21 solitary, 9 multiple	7	Evacuation	-	Biliary fistula 2	1

A total of 11 reports and six techniques addressing hepatic hydatid cysts using specially designed laparoscopic instrumentation were identified. There were 438 patients in total, with the largest group (n=291) benefiting from the Palanivelu approach. This technique is similar to the one presented, with a trocar containing both an aspiration mechanism and cannulation. After puncturing the cyst, the instrumentation creates a suction system that prevents leakage around the trocar. This approach was used in 88.58% of cases for solitary hydatid cysts, with the management of the cyst varying in studies, from cystectomy to simple drainage. No conversions were recorded, but the approach was performed exclusively for selected cases. Postoperative complications occurred in 14.77% of cases, with a higher frequency of bile leak complications (12.37%). The detected recurrence rate was 1.03%.

The Perforator Grinder Aspirator (PGAA) technique predominantly addresses the unique hepatic localizations of the hydatid cyst, with a conversion rate of 12.5% and a recurrence rate of 7.81%. The approach with the Veress needle is limited to cyst drainage, reporting a complication rate of 22.22%. The Hemmati system and port-in-cyst technique were described in a limited case series, predominantly addressing solitary cysts and primarily performing drainage, with a proportion of 81.81%. The umbrella trocar technique exclusively evacuates the content of the hydatid cyst, with a conversion rate to open surgery of 23.33%, a postoperative complication frequency of 6.66%, and a recurrence rate of 3.33%. Treatment in hepatic hydatid disease aims to eliminate local disease and associated complications, prevent progression to local or systemic complications, and achieve therapeutic management that is as minimally invasive as possible, with minimal intraoperative complications, and low rates of postoperative mortality and morbidity.

The intraperitoneal or systemic dissemination of this disease most commonly results from the rupture of the hydatid cyst, either spontaneously, following trauma, or post-interventionally. Spontaneous cyst rupture is typically caused by increased intracystic pressure or cysts larger than 10 cm in diameter. Dissemination of cyst contents can occur during interventional approaches such as PAIR, modified PAIR, or Modified Catheterisation Technique (MoCAT), as well as during surgical approaches, whether laparoscopic or open surgery. The result is manifested by the appearance of intraperitoneal localizations, abdominal hydatidosis, or recurrences [25]. The success of managing this disease is gauged by a low recurrence rate or absence of recurrences. Despite protective measures through pre- and postoperative treatment, minimally invasive approaches, parasitic inactivation solutions, and intraoperative protective methods, the recurrence rate of hepatic hydatid cysts ranges between 4.6% and 22% [25].

The main causes of recurrence are intraoperative spillage of active, viable cyst content; failure to remove the cysts in difficult locations; or retention of viable cystic fragments in the cyst wall. The latter situation is encountered in conservative approaches to the cyst wall. Recurrence in the immediate postoperative period is usually caused by an inefficient primary partial surgical approach. The solution would be a more radical approach, but this comes with more frequent intra- and postoperative complications, longer operative duration, and increased conversion rate to open surgery. The controversy surrounding open surgery vs. laparoscopy in hepatic hydatid cyst surgery has been settled, with the laparoscopic approach now considered superior to open surgery in all aspects except for the recurrence rate. The open approach is still preferred in cases of multiple intra-abdominal localizations, difficult intrahepatic localizations, or those in contact with major vascular or biliary elements, as well as a backup solution in the laparoscopic approach [26, 27].

While PAIR is superior to the surgical approach in terms of postoperative complications, aesthetic benefits, and patient recovery, it is deficient in terms of recurrence. This is attributed to a deficit in isolating the cyst and limiting the risk of spillage [28]. Interventions with a conservative approach to the cyst wall, whether in the form of PAIR or laparoscopy, despite being more prone to recurrence, represent the main method of managing the condition due to their benefits to the patient, even if they are burdened by a higher recurrence rate.

A therapeutic solution in surgery involves an approach to hydatid disease with instrumentation designed to prevent situations leading to intraoperative spillage or recurrence. Although not very common, each presents characteristics that aim to fulfill the attributes of safe hydatid surgery. Comparing these techniques, it is observed that they were most commonly used for single localizations of hydatid disease and in selected cases. Moreover, the predominant approach to the cystic cavity and cyst wall was a conservative one, with the Palanivelu technique and the approach used by our team being the only ones used to perform excisional procedures on a larger scale. Approaches such as umbrella trocar, port-in-cyst, or Veress needle, cannot perform excisional procedures but only inactivation and evacuation of the hydatid content, due to their design. PAIR procedures achieve the same steps but in a less invasive manner. The higher rates of conversion, postoperative complications, and recurrence make some of the described procedures less effective compared to other techniques using specialized instrumentation, and even compared to standard laparoscopic or open surgery approaches.

Our study revealed that the approach with dedicated instrumentation was equally performed for both single and multiple cysts. The procedure was also applied to unselected cases, emergencies, and cases with local complications (such as penetration into the diaphragm, gallbladder involvement, and abscesses), and the overall conversion rate was significantly lower compared to the laparoscopic group. The patented procedure was superior to laparoscopic surgery regarding operative duration, the number of intraoperative complications, and the frequency of radical procedures such as pericystectomy. Better results were also recorded regarding the number of postoperative complications and length of hospital stay. No recurrences were identified. The treatment of hepatic hydatid disease remains a challenge, both in terms of antiparasitic therapy, scolicidal solutions, and invasive procedures.

Pharmacological treatment and scolicidal solutions, although associated with increased effectiveness, cannot achieve complete resolution of hydatid disease. Invasive treatments such as PAIR or laparoscopy achieve inactivation, evacuation, and, if necessary, excision of the cyst, and have the highest therapeutic efficacy, but are burdened, albeit to a small extent, by the risk of spillage and recurrence. These weaknesses are addressed by laparoscopic techniques where the instrumentation is specially designed for hydatid pathology. Robotic surgery for hydatid cysts is still in its early stages of development but has shown promising results; however, definitive conclusions cannot yet be drawn given the lack of extensive studies.

The superiority of laparoscopy over open surgery is unequivocal. The minimally invasive approach brings undeniable benefits in terms of postoperative recovery, aesthetics, duration of hospitalization, costs, and postoperative complications. Minimally invasive access is not limited solely to pathologies common in clinical practice but yields superior results even in unusual cases. Laparoscopic diagnosis and treatment have now become the standard of care for the majority of surgical patients. Hydatid cysts, once exclusively treated with open surgery, now primarily benefit from a laparoscopic approach.

Limitations of the study

The single-center design of our study limits the generalizability of its findings, as they may not accurately represent the wider population. Success rate may be dependent on the learning curve, and limited to surgeons in facilities where this approach is utilized.

## Conclusions

Hydatid cyst is a complex pathology and a public health issue due to its allergenic characteristics, risk of dissemination, as well as local complications, and recurrent nature. Laparoscopic surgery has paved the way for minimally invasive approaches in the therapeutic management of hepatic echinococcosis, with the introduction of specific surgical techniques and instrumentation representing an upgrade with clear advantages for both the surgeon and the patient. The approach and instrumentation studied represent a valid option in the treatment of hydatid cysts in both adults and children and have been the primary technique used for this pathology in our department for almost two decades.
